# Mice expressing the variant rs1143679 allele of *ITGAM* (CD11b) show impaired DC-mediated T cell proliferation

**DOI:** 10.1007/s00335-019-09819-y

**Published:** 2019-10-31

**Authors:** Justin T. Avery, Rachel V. Jimenez, Joseph L. Blake, Tyler T. Wright, Beatriz Leόn-Ruiz, Trenton R. Schoeb, Alexander J. Szalai, Daniel C. Bullard

**Affiliations:** 1 Department of Genetics, University of Alabama at Birmingham, 1700 University Blvd., Birmingham, AL 35294-0013 USA; 2 Department of Medicine, University of Alabama at Birmingham, Birmingham, AL USA; 3 Department of Clinical and Health Sciences, University of Alabama at Birmingham, Birmingham, AL USA; 4 Department of Microbiology, University of Alabama at Birmingham, Birmingham, AL USA

## Abstract

Genome-wide association studies (GWAS) and functional genomic analyses have implicated several *ITGAM* (CD11b) single-nucleotide polymorphisms (SNPs) in the development of SLE and other disorders. *ITGAM* encodes the α_M_ chain of the β_2_ integrin Mac-1, a receptor that plays important roles in myeloid cell functions. The *ITGAM* SNP rs1143679, which results in an arginine to histidine change at amino acid position 77 of the CD11b protein, has been shown to reduce binding to several ligands and to alter Mac-1-mediated cellular response in vitro. Importantly, however, the potential contribution of this SNP variant to the initiation and/or progression of immune and inflammatory processes in vivo remains unexplored. Herein, we describe for the first time the generation and characterization of a mouse line expressing the 77His variant of CD11b. Surprisingly, we found that 77His did not significantly affect Mac-1-mediated leukocyte migration and activation as assessed using thioglycollate-induced peritonitis and LPS/TNF-α-induced dermal inflammation models. In contrast, expression of this variant did alter T cell immunity, as evidenced by significantly reduced proliferation of ovalbumin (OVA)-specific transgenic T cells in 77His mice immunized with OVA. Reduced antigen-specific T cell proliferation was also observed when either 77His splenic dendritic cells (DCs) or bone marrow-derived DCs were used as antigen-presenting cells (APCs). Although more work is necessary to determine how this alteration might influence the development of SLE or other diseases, these in vivo findings suggest that the 77His variant of CD11b can compromise the ability of DCs to induce antigen-driven T cell proliferation.

## Introduction

Mac-1 (*ITGAM/ITGB2*, CD11b/CD18), a member of the β_2_ integrin family of adhesion molecules, is expressed primarily by cells of the myeloid lineage (Tan 2012). ICAM-1, iC3b, RAGE, and CD40L are just a few of its more than 50 biologically important ligands (Rosetti and Mayadas 2016). This adhesion molecule plays an integral role in the recruitment and activation of neutrophils, monocytes, and macrophages during inflammation and participates in phagocytosis and cytokine production (Fagerholm et al. 2013; Herter and Zarbock 2013; Lim and Hotchin 2012). Additional evidence strongly suggests Mac-1 can modulate the immune response, either promoting or inhibiting it depending on the context (Ehirchiou et al. 2007; Han et al. 2010; Ren et al. 2004). For example, engagement of Mac-1 on certain subsets of dendritic cells (DCs) can, depending on the context, either amplify or restrict their ability to stimulate T cell proliferation (Behrens et al. 2007; Chen et al. 2008; Monrad and Kaplan 2007; Sandor et al. 2013; Schmidt et al. 2006; Skoberne et al. 2006; Varga et al. 2007). Thus, while splenic DCs isolated from *Itgam* null mice (mutants that lack expression of CD11b) showed an impaired ability to promote T cell proliferation (Ling et al. 2014), bone marrow-derived dendritic cells (BMDCs) from the same mutants had a decreased capacity to suppress T cell proliferation (Bai et al. 2012). Likewise, loss or inhibition of Mac-1 has been reported to protect from or exacerbate inflammation and autoimmunity in different murine models, suggesting a complicated immunoregulatory role for this integrin in disease development (Bullard et al. 2005; Kevil et al. 2004; Leon et al. 2006; Soloviev et al. 2011; Stevanin et al. 2017).

Genome-wide association studies (GWAS) have now linked several *ITGAM* single-nucleotide polymorphisms (SNPs) with the risk and severity of disorders such as systemic lupus erythematosus (SLE), systemic sclerosis, and melanoma (Anaya et al. 2012; Hom et al. 2008; Lenci et al. 2012; Nath et al. 2008). One of these *ITGAM* variants, the non-synonymous SNP rs1143679 that results in a 77Arg → 77His change in the extracellular domain of human CD11b, has been shown to modulate certain Mac-1-mediated functions in vitro. For example, cell lines transfected to express human CD11b encoding the 77His variant showed impaired phagocytosis and adhesion and increased IL-6 generation compared to control cells expressing the invariant protein, which has an arginine at position 77 (MacPherson et al. 2011). In addition, primary cells (neutrophils and monocytes) from human donors expressing the 77His *ITGAM* variant exhibited reduced adhesion to iC3b, ICAM-1, and fibrinogen, decreased phagocytosis of opsonized particles, and altered cytokine expression (Fossati-Jimack et al. 2013; Reed et al. 2013; Rhodes et al. 2012; Rosetti et al. 2015; Zhou et al. 2013). Each of these findings has to be interpreted with some caution, however, because (i) in transfected cells expressing *ITGAM* variants, the function of CD11b ultimately depends on its ability to pair with CD18, whether expressed naturally or after co-transfection with *ITGB2*; and (ii) in primary cells, the potential impact of other *ITGAM* SNPs known to be in high linkage disequilibrium (LD) with rs1143679 is not always accounted for. Consequently, it might be that not all of the functional defects so far attributed to the rs1143679 variant are due to 77His per se. Indeed, our group showed that the SNP rs1143678, resulting in a proline to serine change at amino acid position 1146, compromises neutrophil adhesion and phagocytosis in the absence of the rs1143679 77His variant (Zhou et al. 2013). Lastly, in general, biologically meaningful effects might occur in vivo that might not be detectable in vitro and vice versa.

We took advantage of the high homology between human *ITGAM* and mouse *Itgam* to assess the impact of the rs1143679 77His variant on Mac-1-dependent processes in vivo. To accomplish this in complete isolation from other potentially confounding *ITGAM* variants in LD, we generated by gene targeting in embryonic stem cells mice expressing histidine at amino acid position 77 of the naturally expressed mouse CD11b protein. In contrast to prior reports of impaired ligand binding, adhesion, and migration of 77His transfected and primary cells in vitro, we found that compared to wild-type controls, mice expressing 77His showed no statistically significant changes in neutrophil and monocyte recruitment or the extent of tissue damage when subjected to acute inflammation models. However, expression of 77His in mice significantly diminished antigen-specific T cell proliferative responses. Importantly, this effect on T cells was also observed ex vivo in co-cultures using primary spleen DCs or bone marrow-derived DCs (BMDCs) from 77His mice as APCs. These are the first experiments to address the impact of 77His in vivo and they establish that expression of this variant form of CD11b can impair the ability of DCs to support a full T cell response in mice. Ongoing studies are aimed at identifying the specific Mac-1-dependent processes altered by 77His that might be responsible for this effect, and determining whether this effect is sufficient to alter the course of T cell-driven autoimmunity.

## Materials and methods

### Structural modeling of the human and murine CD11b proteins

To obtain evidence that the influence on CD11b of introducing the 77His variant should be comparable in the two species, and to thereby validate our genetic engineering approach, structural models of human and murine CD11b were rendered using the COACH meta-server for protein–ligand binding site prediction (Yang et al. 2013a, b). The respective invariant (77Arg in human, 77Pro in mouse) and variant (77His) CD11b structures were predicted using the multi-sources threader (MUSTER) algorithm (https://zhanglab.ccmb.med.umich.edu/MUSTER/) applied to the CD11c template 3k71G obtained from the protein data bank (PDB) library (https://www.wwpdb.org/). Each structure was then rendered and the invariant and variant versions aligned using the DeepView/Swiss PDBViewer v4.1.0 (https://spdbv.vital-it.ch/).

### Generation of the 77His mouse line

The *Itgam* variant mouse line (C57BL/6 J) encoding histidine at amino acid position 77 (77His mice) was generated using an embryonic stem cell gene-targeting strategy as outlined in the *Results* section. Both conventional sequencing and pyrosequencing of genomic DNA isolated from tail biopsies were used to confirm the presence of the anticipated C to A nucleotide substitution in exon 3 of the targeted *Itgam* gene. Pyrosequencing was performed using the following primers: forward—GACAGGTGCCCTCTACCAGTG, reverse—TTGACAAGCCAGGGGTGTTCAC, and sequencing—TGAGACTCACCTTGCAG. To confirm the C to A nucleotide exchange in *Itgam* transcripts, total RNA isolated from the spleens of heterozygous (CA) and homozygous (AA) mice was used as a template for the generation of cDNA (forward primer—TGGTCCAGCTTGGCGGAACC, reverse primer—CCTCTGGGAACTGCTGGGGC), cloned into the TOPOTA cloning vector (ThermoFisher Scientific), and then sequenced.

C57BL/6 J wild-type (WT) and flippase (FLP) mice (B6.129S4-Gt(ROSA)26Sortm1(FLP1)Dym/RainJ) were purchased from The Jackson Laboratory (Bar Harbor, ME). OT-II/CD45.1 mice were generated by crossing OT-II mice (B6.Cg-Tg[TcraTcrb]425Cbn/J) expressing a TCR-specific for ovalbumin (OVA) residues 323-339 [provided by Dr. Francis R. Carbone (Barnden et al. 1998)] with CD45.1 mice expressing the Ptprc^a^ allele on the C57BL/6 background (B6.SJL-*Ptprc*^*a*^*Pepc*^*b*^/BoyJ; The Jackson Laboratory). Mice containing a CD11b null mutation were generated as previously described (Lu et al. 1997). This mutation was backcrossed for 11 generations onto the C57BL/6 J strain and homozygotes were generated by intercrossing. All animal procedures including euthanasia were approved by The University of Alabama at Birmingham Institutional Animal Care and Use Committee.

### Antibodies and flow cytometry

The following fluorochrome-conjugated anti-mouse antibodies (clones) were used: anti-CD11b (M1/70), -CD11c (N418), -CD45.1 (A20), -CD80 (16-10A1), -CD86 (GL-1), and -CD4 (RM4-5) all from BioLegend (San Diego, CA); anti-GR1 (RB6-8C5), -Ly6C (HK1.4), -CD3e (145-2C11), and -CD28 (37.51) all from eBioscience (San Diego, CA); and anti-MHC-II (M5/114.15.2) from BD Bioscience (San Jose, CA). LIVE/DEAD stain was used to determine the viability of cells and was purchased from Life Technologies (Eugene, OR). Flow cytometry was performed using an LSRII instrument and the acquired data were analyzed using FlowJo software.

### Peritonitis model

Age- and sex-matched 77His and WT mice were injected i.p. with 1 mL of sterile 4% thioglycollate (T9032, Sigma Aldridge) or 1 mL of tissue culture grade PBS as a control. At 4, 24, or 48 h thereafter, mice were euthanized and 10 mL PBS was introduced into the peritoneal cavity. The resultant lavage fluid was collected and peritoneal exudate cells were harvested, counted, labeled using anti-CD11b, -Ly6c, and -GR1, and subjected to flow cytometry to enumerate neutrophils (CD11b^+^/Ly6C^+^/GR1^high^ cells) and monocytes/macrophages (CD11b^+^/Ly6C^very high^/GR1^low^ cells).

### Dermal inflammation model

The dermal Shwartzman reaction was induced as described previously (Rothstein and Schreiber 1988). Briefly, age- and sex-matched 77His and WT mice received s.c. 100 μg of lipopolysaccharide (LPS from *Escherichia coli* serotype 055:B5; Sigma-Aldrich) in PBS (100 μl). Twenty-four hours later (day 1), recombinant TNF-α (0.3 μg/mouse; aa 80-235, R&D Systems) was injected s.c. at the same site. Control mice received two injections of PBS. Mice were euthanized 24 h later (day 2) and the skin at the injection site was excised, fixed in buffered 10% formalin/70% ethanol, and processed into paraffin. Thin sections (5 µm) were prepared and stained with hematoxylin and eosin (H&E) for microscopic evaluation, and neutrophilia, hemorrhage, and edema were then scored for their extent and severity in a blinded fashion. For each thin section inspected, the extent of each measure was scored 0 for absent, 1 for > 0–25%, 2 for 25–50%, 3 for 50–75%, and 4 for 75–100% affected. The severity of each measure was scored 1, 2, or 3 for mild, moderate, and severe, respectively, with increments of 0.5 for intermediate degrees (e.g., 2.5 for moderate to severe). The score was calculated as extent **×** severity. Scores for each section were averaged. Vasculitis was scored 1, 2, or 3 for mild, moderate, and severe, respectively, with increments of 0.5 for intermediate degrees for each affected vessel. The number of affected vessels having thrombi was counted. The *overall score* for each mouse was calculated as (neutrophil extent **×** severity) + (hemorrhage extent **×** severity) + (edema extent **×** severity) + sum of vasculitis scores + count of thrombi. Vasculitis severity also was assessed separately by averaging the individual vasculitis scores for each mouse.

### In vivo T cell proliferation

OT-II/CD45.1 mice were euthanized and their spleens and lymph nodes collected. These were disrupted and after RBC lysis (Sigma-Aldrich, St. Louis, MO) CD4^+^ T cells were isolated by negative selection using the mouse CD4^+^ T cell isolation kit 19852A (Stemcell Technologies; Seattle, WA) and labeled with Celltrace CFSE (Invitrogen; Eugene, OR). 1 **× **10^7^ CFSE-labeled T cells were injected into the tail vein of 77His or WT mice (day 0), and 24 h later (day 1), each mouse received i.v. 100 μg OVA (A-5503; Sigma Aldridge). On day 4, mice were euthanized and their spleens were harvested. Spleen cells were isolated and labeled with anti-CD4 and anti-CD45.1 antibodies, and CD4^+^CD45.1^+^CFSE^+^ OT-II T cells (events) identified by flow cytometry. To determine the percentage of T cells present in each proliferating generation, the following equation was used:

$$\frac{{{\text{Events in Generation}} \times 2^{\text{Generation Number}} }}{{\sum {\text{Transformed Events}}}}$$

### In vitro T cell proliferation assays

Both splenic DCs (CD11c^+^ cells) and bone marrow-derived dendritic cells (BMDCs) from 77His and WT mice were used as APCs for in vitro T cell proliferation assays. Splenic DCs were isolated from spleens by positive selection using the EasySep Mouse CD11c^+^ Selection Kit II (18780A; Stemcell Technologies, Seattle, WA), and CD4^+^ CD45.1^+^ CFSE^+^ OT-II T cells were isolated as described above. 2 **× **10^6^ CFSE-labeled T cells were co-cultured with 4 **× **10^5^ DCs (77His, WT, or CD11b−/−) in 96-well round-bottom plates in RPMI complete medium (RPMI 1640, 5% FBS, 2 mM L-GlutMAX, 50,000 U penicillin/streptomycin, 1% non-essential amino acids, 50 µM β-mercaptoethanol). Seventy-two or 120 h after supplementation with OVA_323–339_ peptide (1.0 µM; New England Peptide, Gardner, MA), T cell proliferation (CFSE signal diminution) was assessed by flow cytometry and the percentage of T cells present in each proliferating generation was then determined using the formula described above for the in vivo T cell proliferation analyses.

BMDCs were generated as previously described (Jimenez et al. 2018). Briefly, bone marrow was flushed from tibias and femurs of 77His and WT mice and 1 **× **10^6^ cells were plated in 12-well flat-bottom plates and cultured in RPMI 1640 media supplemented with 5% FBS, 2 mM L-GlutaMAX, 50,000 U penicillin/streptomycin, 1% non-essential amino acids, 50 mM β-mercaptoethanol, and 20 ng/mL granulocyte–macrophage colony-stimulating factor (GM-CSF). The medium was changed on days 3 and 5. For antigen-specific T cell proliferation assays, BMDCs were stimulated/matured with 10 μg/mL LPS in the absence or presence of different concentrations of OVA_323–339_ peptide (10, 20, 40, and 60 nM) on day 6. On day 7, BMDCs were harvested and used in co-culture assays with 2 × 10^5^ CFSE-labeled CD4^+^CD45.1^+^ OT-II T cells. Cells were cultured at a 1:5 BMDC:T cell ratio in 96-well plates for 72 h, and T cell proliferation was measured by flow cytometry (CFSE diminution).

### Analysis of costimulatory markers

WT and 77His mice were euthanized and their spleens were removed. After RBC lysis, spleen cells were incubated in medium containing RPMI 1640, 5% FBS, 2 mM L-GlutaMAX, 100 μg/mL pen/strep, 1X non-essential amino acids, and 55 μm β-mercaptoethanol (Fisher Scientific, Lenexa, KS). Cells were then treated with 1 μg/mL LPS or left untreated for 24 h, fixed in 4% paraformaldehyde, incubated with Fc block (BD Biosciences), and labeled with anti-CD11b, -CD40, -MHC-II, -CD80, -CD86, and -CD11c antibodies, and the expression of these markers were analyzed by flow cytometry.

### Statistical analyses

Where appropriate, the data are presented as means ± their standard deviations. Differences between genotypes and treatments were assessed using Student’s *t* tests or one-way ANOVA with post hoc Tukey’s multiple comparisons tests. Non-parametric measures were assessed using the Mann–Whitney U test. Differences yielding a *p* value ≤ 0.05 were considered significant in all statistical analyses, which were performed using GraphPad Prism 7 software (Graphpad, La Jolla, CA).

## Results

### A predicted minor effect of the 77His substitution on the structure of human CD11b is reproduced in the structurally homologous mouse CD11b protein

**Fig. 1:**
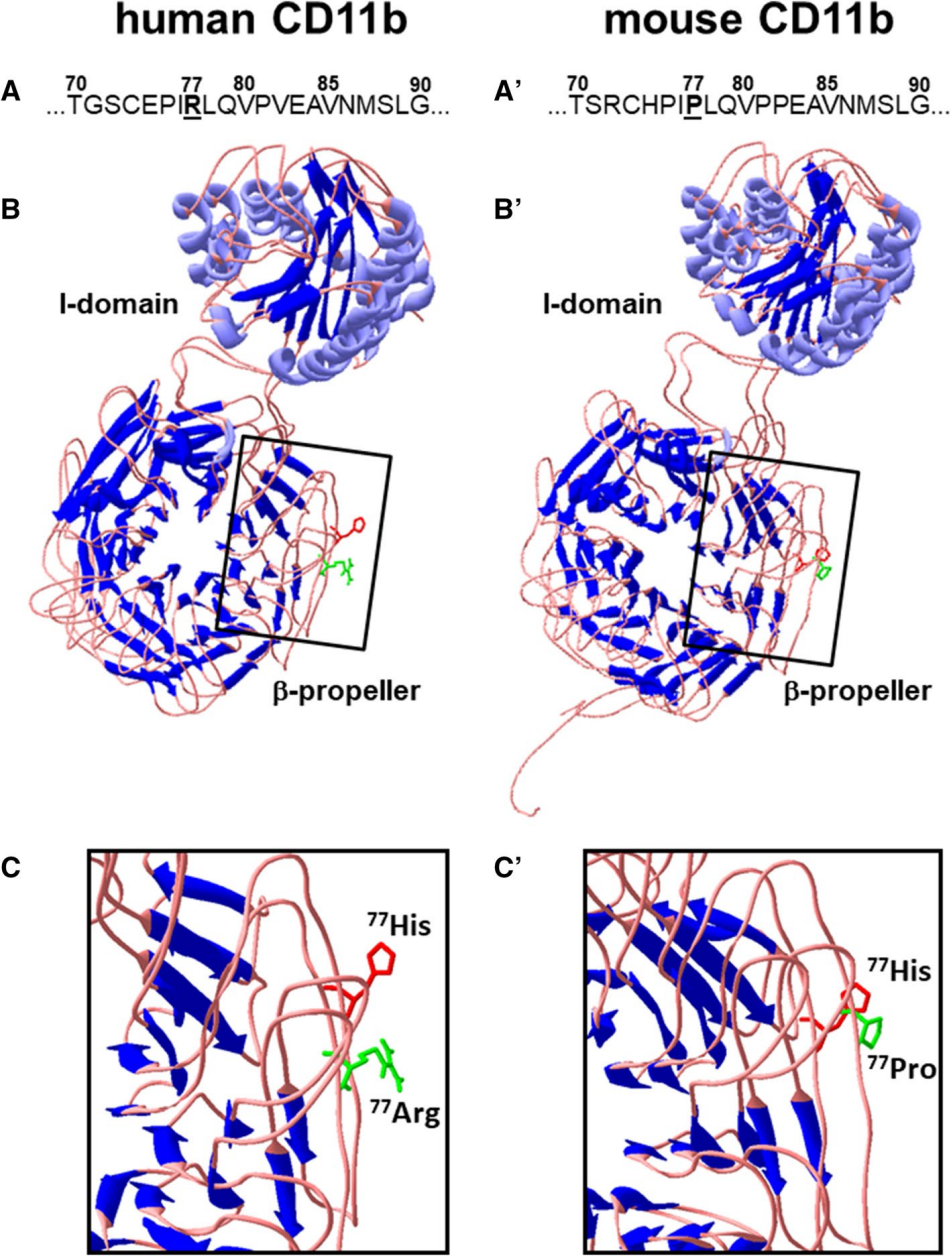
Amino acid sequences and predicted structures of human and mouse CD11b and their respective 77His variants. **a** Amino acid sequence of the region of human CD11b adjacent to position 77 (the underlined bold residue). **b** The predicted structure of the human CD11b I-domain and β-propeller regions, with the predicted structures for the common 77Arg (green) and the rs1143679 SNP variant 77His (red) proteins overlaid. **c** The boxed area in B is expanded to show the relative positions of the human 77Arg versus 77His residues. **a′**–**c′** The wild-type (77Pro; green) versus the engineered (77His; red) versions of the analogous regions of mouse CD11b are shown. In all four situations, residue 77 is in an exposed position on the flank of the β-barrel, which might allow it to contribute to interactions with Mac-1 ligands. Tertiary structures were predicted with MUSTER using the 3k71G structure of CD11c as a template

Published studies using human leukocytes and transfected cell lines suggest that the 77His *ITGAM* variant significantly inhibits or alters Mac-1-dependent functions like adhesion, phagocytosis, and cytokine production (Fossati-Jimack et al. 2013; MacPherson et al. 2011; Maiti et al. 2014; Reed et al. 2013; Rhodes et al. 2012; Rosetti et al. 2012, 2015; Zhou et al. 2013). While these studies have been very informative, they cannot reveal how the 77His variant contributes to authentic immune and inflammatory processes since in vitro and ex vivo systems cannot recapitulate the different tissue microenvironments and biological stimuli encountered by leukocytes in vivo, nor can they effectively model all of the potential cellular interactions, trafficking patterns, and antigens present in the whole organism. To directly address this problem, we took advantage of the high homology between human *ITGAM* and mouse *Itgam* and developed a line of mice to investigate the in vivo impact of the rs1143679 variant allele on Mac-1-dependent responses. We first performed structural analyses to compare the predicted effects of the 77His substitutions on the conformation of the extracellular domain of the human and mouse CD11b proteins. Human *ITGAM* and mouse *Itgam* are highly homologous; they share 78.7% DNA sequence similarity and their encoded proteins have 74.8% direct amino acid identity. Importantly, this high homology is retained in the region that includes the 77His variant (Fig. 1; compare the sequences shown in panels A and A’). Protein modeling was used to render a predicted structure of the human and mouse CD11b β-propeller and I-domain regions (Fig. 1b and b′, respectively). Comparison of the predicted structures of the human invariant (77Arg) and variant (77His) proteins revealed only subtle changes (Fig. 1b and c). The predicted structure of the same region in mouse CD11b (Fig. 1b′) was very similar to that in human CD11b and, as for the human variant, introduction of a histidine at position 77 caused only subtle changes in mouse CD11b (Fig. 1c′). In all four situations, residue 77 is in an exposed position on the flank of the β-barrel, which might allow it to contribute to interactions with Mac-1 ligands.

### Generation of the 77His mouse line

**Fig. 2:**
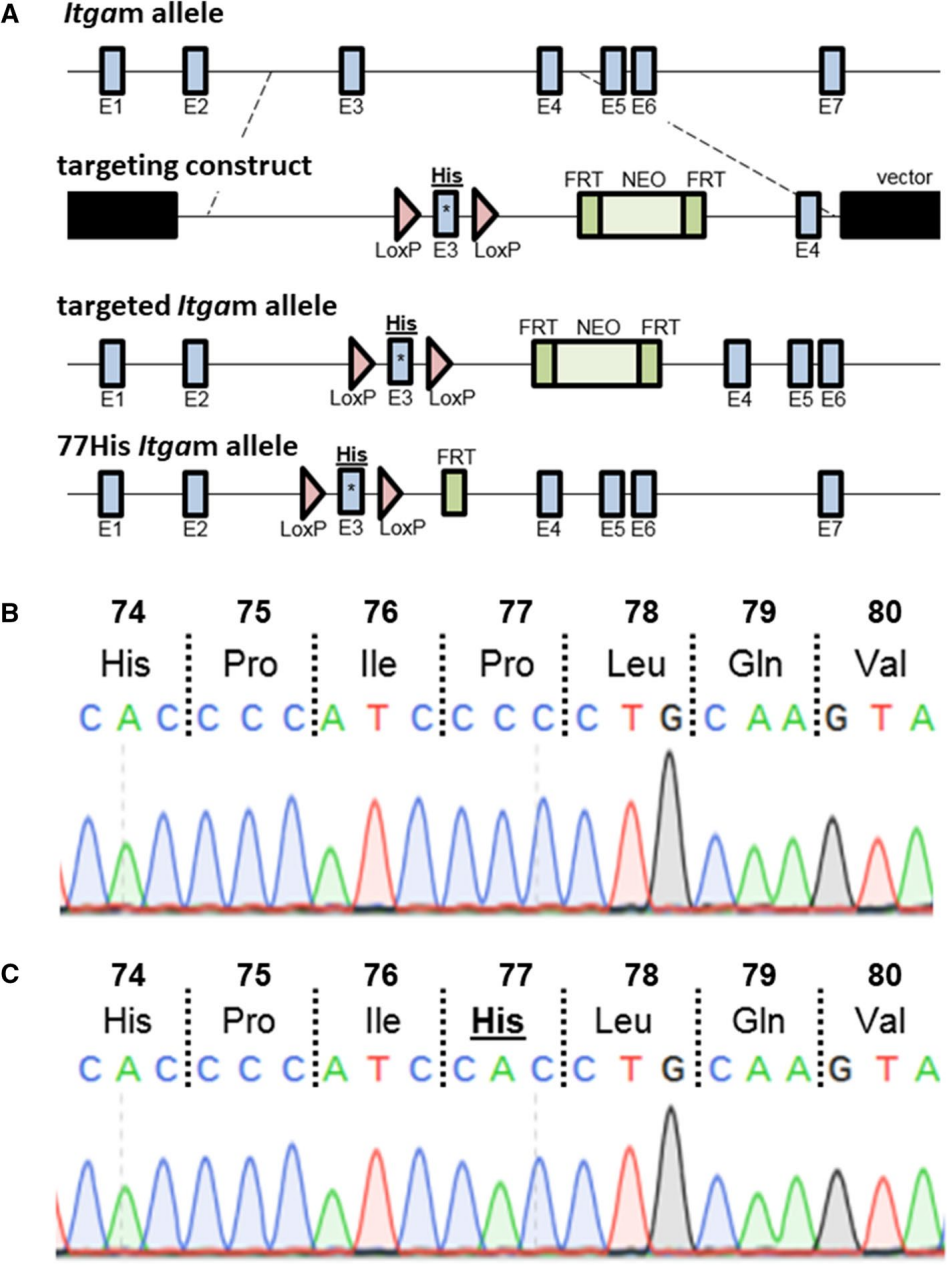
Generation of the 77His variant C57BL/6 mouse. **a** The wild-type *Itgam* allele was targeted using a replacement construct that included *LoxP* sites to flank exon 3 (E3), wherein the 77His variant is encoded. The neomycin selection gene (NEO; located between E3 and E4 and flanked by FRT recognition sites) was removed by breeding founder mice to *FLP* recombinase-expressing mice. This yields the 77His *Itgam* allele. **b** DNA sequencing trace for a WT cDNA clone derived from the spleen of a 77His heterozygous mouse; note that Pro is encoded at position 77. **c** DNA sequencing trace for a variant cDNA clone derived from the spleen of a 77His homozygous mouse; note that His is encoded at position 77

**Fig. 3:**
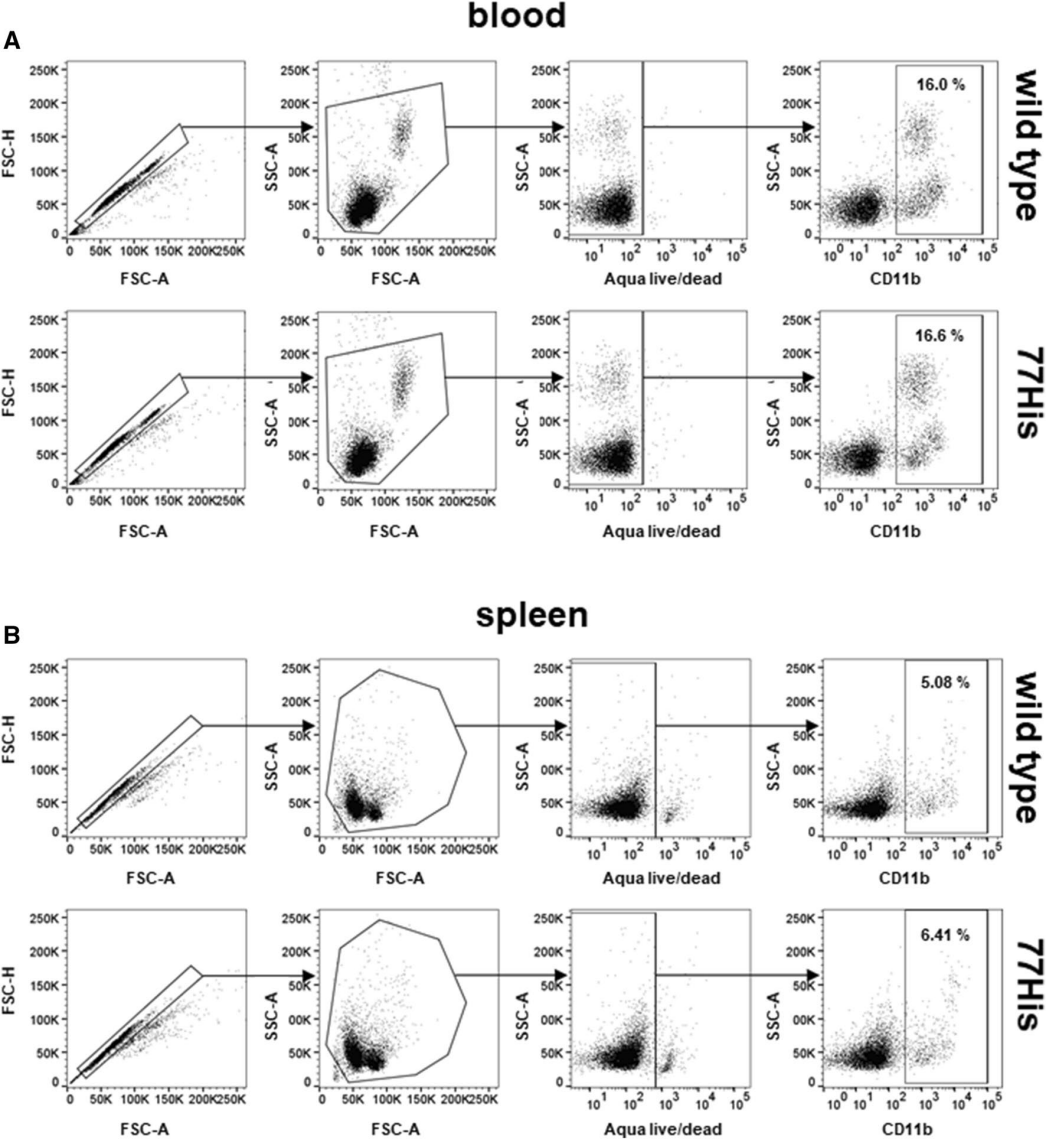
Mac-1 expression by circulating and tissue resident myeloid cells in WT and 77His mice. Mac-1 expression was analyzed by flow cytometry of peripheral blood cells (**a**) and spleen cells (**b**) obtained from 77His and WT mice. In each instance, single cells were identified by FSC-A vs FSC-H, and cells of interest were gated by FSC-A vs SSC-A, and live cells (aqua Live/Dead negative) were analyzed. There was no difference in the proportion of CD11b^+^ cells detected (percentages shown) or the level of CD11b expression (not shown) between genotypes

Our structural modeling suggested that the single 77Pro → 77His substitution in the mouse CD11b protein should not lead to any major tertiary structural changes that could potentially render Mac-1 incapable of interacting with its natural ligands. Accordingly, by gene targeting in embryonic stem cells, we generated mice expressing histidine at amino acid position 77 of the naturally expressed mouse CD11b. This strategy has the added advantage of allowing us to study the impact of 77His in complete isolation from other potentially confounding *ITGAM* variants known to be in high LD. We designed a replacement construct (Fig. 2a) to change the proline expressed at position 77 in WT CD11b to a histidine, thereby mimicking the human rs1143679 SNP variant. In addition to the intended sequence change, the targeting construct included (i) flanking *FLP* recombinase recognition target (*FRT*) sites for subsequent removal of the *neomycin* selection gene and (ii) *LoxP* sites to generate a conditional *Itgam* allele for future studies. The targeting construct was introduced into C57BL/6 ES cells by electroporation, and correctly targeted clones (identified by PCR) were injected into blastocysts. After confirmation of germline transmission, mice with the correct nucleotide substitution (*i.e.,* C to A in codon 77 of the mature CD11b protein; see Fig. 2c) were identified by genomic sequencing of the exon 3 region. Heterozygotes were then crossed to mice expressing *FLP* recombinase. Their resulting 77His progeny, lacking the neomycin gene, were intercrossed to generate 77His homozygotes. We confirmed that the C to A nucleotide substitution was transcribed with high fidelity by sequencing cDNA clones generated from splenic mRNA of 77His heterozygous and homozygous mice (Fig. 2b and c, respectively), and we verified that Mac-1 expression on peripheral blood leukocytes and spleen cells from 77His mice was comparable to those from WT (Fig. 3a and b, respectively). To date, there has been no evidence of embryonic lethality or obvious visible phenotypes observed in the 77His mice.

### 77His does not affect myeloid cell recruitment during localized inflammation

**Fig. 4:**
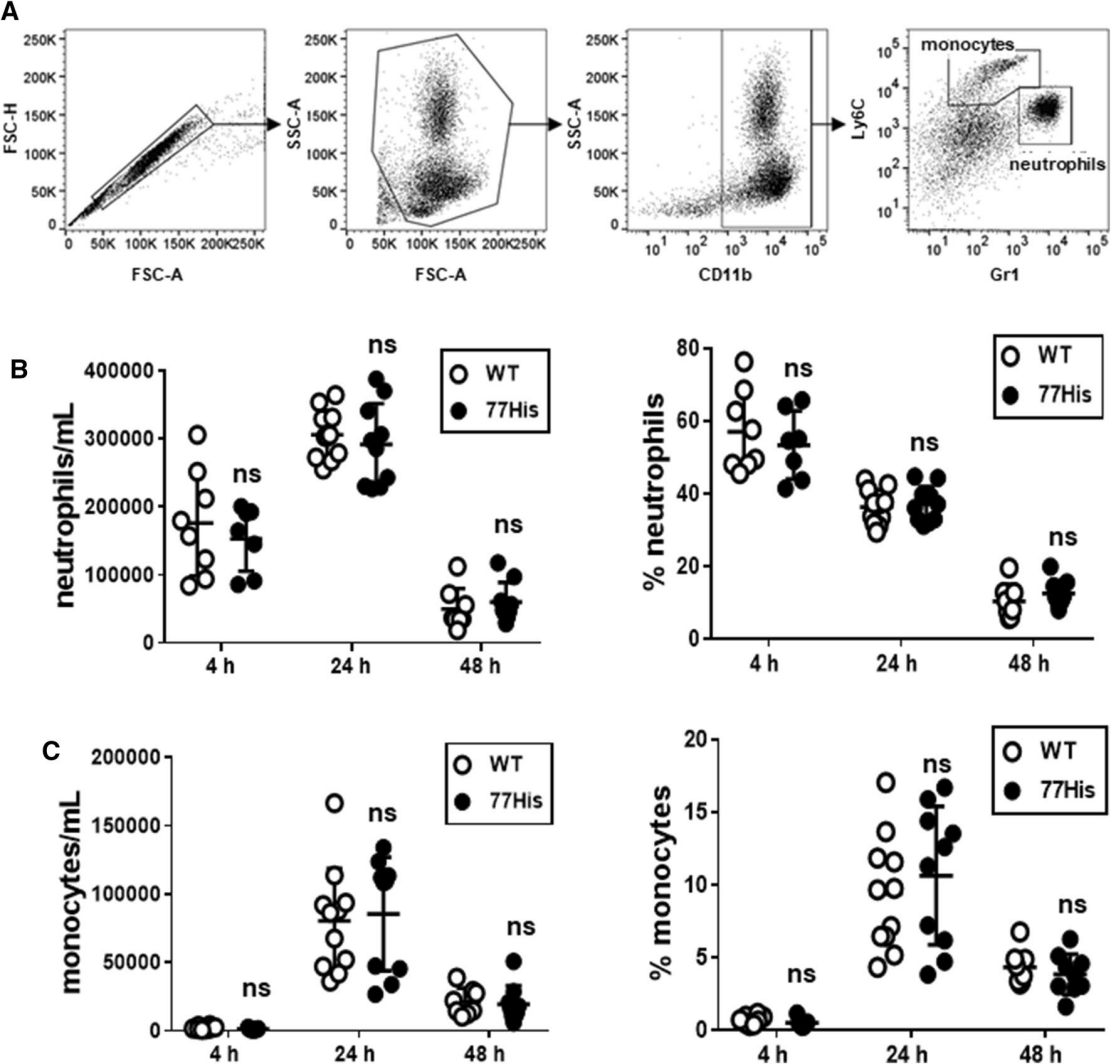
No effect of the 77His variant on thioglycollate-induced peritonitis. **a** Four, 24, or 48 h after injection of thioglycollate i.p., the peritoneal cavity was lavaged and the fluid collected (see "Methods"). Monocytes (CD11b^+^, GR-1^low^, and Ly-6c^very high^) and neutrophils (CD11b^+^, GR-1^high^, and Ly-6c^+^) were enumerated by flow cytometry. The infiltration of the peritoneal cavity by neutrophils (**b**) and monocytes (**c**) was unaffected by 77His. The data shown are the mean ± standard deviation for *N* = 7–11 mice per time point per genotype. ‘ns’ indicates not significant (*p* > 0.05) for *t* tests comparing 77His to time-matched WT

**Fig. 5:**
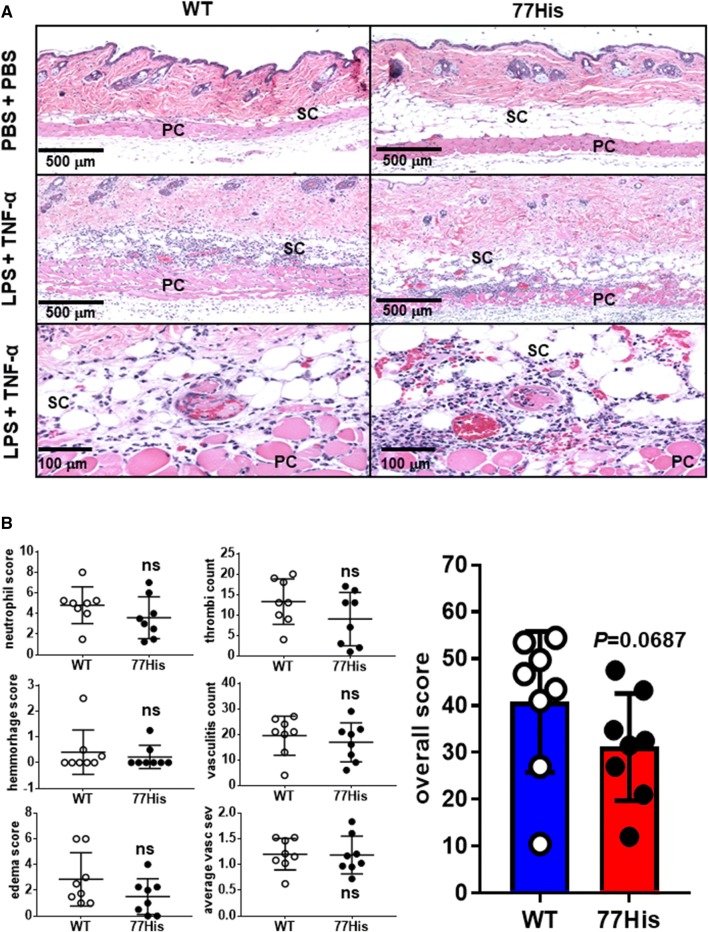
Marginal effect of the 77His variant in the dermal Shwartzman reaction. **a** Mice were injected sequentially with LPS and (24 h later) TNF-α as described in the "Methods"; controls were twice injected with PBS. Twenty-four hours after the last injection, skin biopsies were collected, processed, and stained with H&E for assessment of tissue pathology. Representative thin sections (5 µm thick) from WT (left panels) and 77His mice (right panels) are shown. In LPS + TNF-α-treated mice of both genotypes, significant neutrophil recruitment in the subcutis (SC) and panniculus muscle (PC) was observed, as well as extensive vasculitis and thrombosis. **b** Scores for each individual component of dermal inflammation assessed (see "Methods") were not significantly different (ns) between 77His versus WT mice. The overall score for dermal inflammatory changes was reduced in 77His compared to wild type, but this difference also did not achieve statistical significance (the *p* value for a Mann–Whitney test is given). The mean ± standard deviation is shown for *N* = 8 mice per genotype

The importance of Mac-1 in the recruitment of neutrophils and monocytes to sites of inflammation has been well established (Coxon et al. 1996; Dirk Nolte et al. 1994; Jawhara et al. 2017; Mulligan et al. 1998; Tomasz Liberek et al. 2004), and ex vivo studies using human neutrophils and monocytes expressing 77His showed impairment of Mac-1 binding to ligands such as ICAM-1 (Rhodes et al. 2012; Rosetti et al. 2015; Zhou et al. 2013). To determine whether recruitment of neutrophils and monocytes in vivo was affected by 77His, we subjected mice to two different models of sterile localized inflammation. First, we induced peritonitis by injecting thioglycollate. Leukocytes were isolated from the peritoneum of 77His and WT mice by lavage at 4, 24, and 48 h, stained with antibodies for CD11b, Ly6c, and GR1, and neutrophils and monocytes enumerated by flow cytometry as shown in Fig. 4a. 77His and WT mice were also injected with PBS as a control, which did not promote leukocyte recruitment into the peritoneal cavity at any of these time points (data not shown). We observed no significant differences in either the concentration or the frequency of either cell type recruited to the body cavity in 77His versus WT mice (Fig. 4b and c, respectively). We next compared the responses of 77His versus WT mice subjected to the Shwartzman reaction model of localized skin inflammation (Rothstein and Schreiber 1988). In this model, injection of LPS and TNF-α upregulates ICAM-1 and activates complement, thereby generating the Mac-1 ligand iC3b which, together with cytokine/chemokine production, promotes neutrophil recruitment to the site of injection. The recruited neutrophils subsequently degranulate, leading to fibrin deposition and hemorrhage (Hirahashi et al. 2006). Accordingly, 24 h after induction of the Shwartzman reaction, skin samples (Fig. 5a) were collected and neutrophil infiltration, fibrin deposition, hemorrhage/edema, and vasculitis were assessed. Although there was a distinct trend towards diminished overall scores for the Shwartzman reaction in 77His mice compared to WT (Fig. 5b), neither this difference nor the differences in each component of the overall score achieved statistical significance. The results of the two studies indicate that the 77His variant has little or no impact on leukocyte recruitment after thioglycollate or LPS/TNF-α induced inflammation.

### Dendritic cell function is impaired in 77His mice

**Fig. 6:**
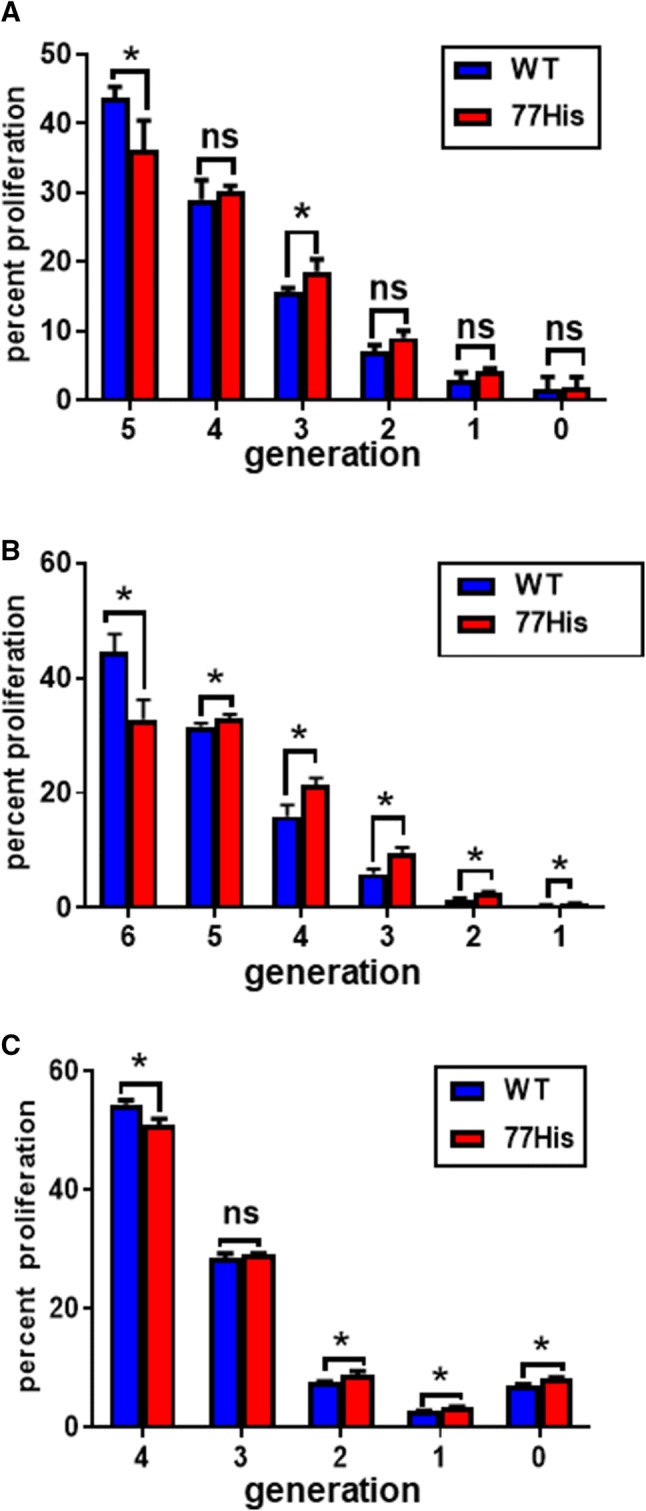
77His compromises antigen-driven T cell proliferation in vivo in mice and in vitro in splenic DC/T cell cultures. **a** WT and 77His mice received i.v. 1x10^7^ CFSE-labeled CD4^+^CD45.1^+^ OT-II T cells, followed 24 h later by 100 μg OVA (i.v.). After 72 h, spleens were harvested and flow cytometry used to assess the proliferation of donor CFSE^+^ T cells. T cell proliferation was reduced in 77His recipients, as indicated by a significant decrease in the percentage of T cells reaching generation 5. *N* = 3 mice per genotype. Non-parametric Mann–Whitney *U* test; *p* < 0.05 (*). **b** Dendritic cells were isolated from the spleens of 77His and WT mice (see “Methods”) and used as APCs for CFSE-labeled CD4^+^CD45.1^+^ OT-II T cells. Co-cultures were provided with 1.0 µM OVA_323–339_ peptide and T cell proliferation was assessed by flow cytometry after 120 h. T cell proliferation was reduced in co-cultures using 77His DCs compared to WT DCs at generation 6. The data shown are from a single in vitro trial, run in triplicate. Differences between genotypes were assessed using the non-parametric Mann–Whitney *U* test. Asterisks indicate *p* < 0.05 (*). **c** Dendritic cells were isolated from the spleens of 77His and WT mice and used as APCs for CFSE-labeled CD4^+^CD45.1^+^ OT-II T cells as described for panel **b**, but T cell proliferation was assessed by flow cytometry after 72 h. T cell proliferation was reduced in co-cultures using 77His DCs, as indicated by a significant decrease in the proportion of T cells reaching generation 4. Similar results were observed across 3 separate experiments (*N* = 3 mice per genotype for each experiment). Non-parametric Mann–Whitney *U* test; asterisks indicate *p* < 0.05 (*). *Ns* not significant (*p* > 0.05)

Previous studies suggest an important role of Mac-1 in modulating the ability of DCs to promote/regulate T cell responses (Behrens et al. 2007; Chen et al. 2008; Monrad and Kaplan 2007; Sandor et al. 2013; Schmidt et al. 2006; Skoberne et al. 2006; Varga et al. 2007). To assess whether the 77His variant had any effect on antigen-specific T cell proliferation in vivo, 1 **× **10^7^ CFSE-labeled CD4^+^CD45.1^+^CFSE^+^ OT-II T cells (donor cells) were administered i.v. to 77His and WT recipients. Twenty-four hours later, each mouse received i.v. 100 μg of OVA. After 72 h to allow for processing of OVA by recipient APCs and its presentation to donor OT-II T cells, spleens were harvested and the proliferation of the donor T cells was assessed. We reproducibly observed that OT-II T cells recovered from the spleens of 77His mice showed significantly reduced proliferation compared to OT-II T cells isolated from WT spleens (Fig. 6a). This effect was evidenced by a significant decrease in the percentage of OT-II T cells that had undergone five cell divisions in 77His compared to WT recipients. This result suggests that in vivo the single 77His change in CD11b is sufficient to compromise the ability of endogenous APCs to induce antigen-dependent proliferation of administered T cells.

To determine whether the observed reduction in donor T cell proliferation might be due to an effect of 77His on endogenous DCs per se, we isolated primary splenic DCs from untreated 77His and WT mice and used them as APCs in co-culture experiments (Fig. 6b). Co-cultures (splenic DCs plus CFSE-labeled CD4^+^CD45.1^+^CFSE^+^ OT-II T cells) were provided with 1.0 µM OVA_323–339_ peptide and T cell proliferation was assessed after 120 h. We found significant decreases in the percentage of OT-II T cells that had undergone six cell divisions in 77His co-cultures compared to WT. A corresponding increase in the percentage of T cells was also observed at generations 1–5 in 77His co-cultures. We next investigated 77His and WT primary splenic DCs for their ability to promote antigen-specific T cell proliferation at a second time point (72 h of co-culture) (Fig. 6c). We observed a significant decrease in the proportion of OT-II T cells that had undergone 4 cell divisions in the presence of 77His DCs compared to WT. In addition, we saw a corresponding increase in the proportion of T cells that underwent 2 or fewer divisions in 77His co-cultures.

**Fig. 7:**
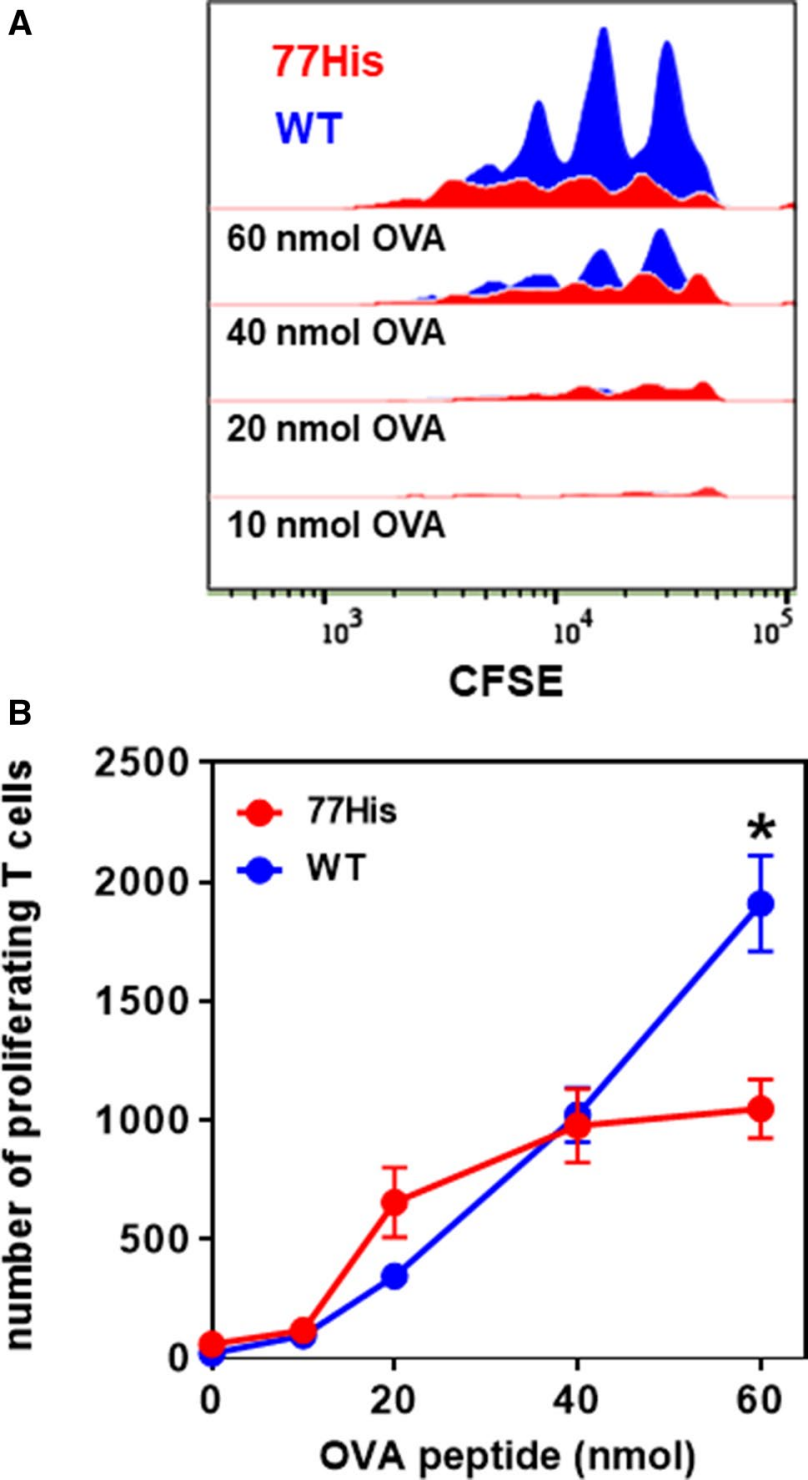
Bone marrow-derived DCs (BMDCs) from 77His mice show impaired capacity to promote antigen-specific T cell proliferation. BMDCs generated from wild-type (WT) or 77His mice were used in co-culture assays to assess their capacity to support antigen-specific proliferation of OT-II cells. **a** Representative CFSE dilution histograms for proliferating CD4^+^ T cells in co-cultures using WT 77His DCs in the presence of increasing amounts of OVA peptide. DCs were incubated with the indicated dose of OVA peptide for 24 h, rinsed, and co-cultured with CFSE^+^ CD4^+^ T cells. After 72 h of co-culture, T cell proliferation was assessed by flow cytometry (CFSE diminution). Note that the proliferation of T cells is reduced in the presence of 77His compared to WT BMDCs. **b** Pooled results (mean ± SEM for *N* = 2 biological replicates with three technical replicates per dose of OVA) from experiments performed as in panel A. One-way ANOVA with post hoc Tukey’s multiple comparisons tests; the asterisk indicates *p* < 0.0001 for WT versus 77His at the indicated dose

To further confirm these findings we next analyzed 77His BMDCs for their ability to promote antigen-specific T cell proliferation. LPS-stimulated 77His and WT BMDCs were incubated with varying concentrations of the OVA peptide for 24 h and then co-cultured with CFSE-labeled CD4^+^CD45.1^+^ OT-II T cells for 72 h (see "Materials and Methods"). In these assays too we observed an antigen concentration-dependent reduction in the number of proliferating T cells in co-cultures using 77His versus WT BMDCs (Fig. 7). The combined results show that the solitary 77His substitution in the CD11b protein is sufficient to compromise the ability of APCs in general, and DCs in particular, to promote antigen-dependent T cell proliferation in vivo and ex vivo.

**Fig. 8:**
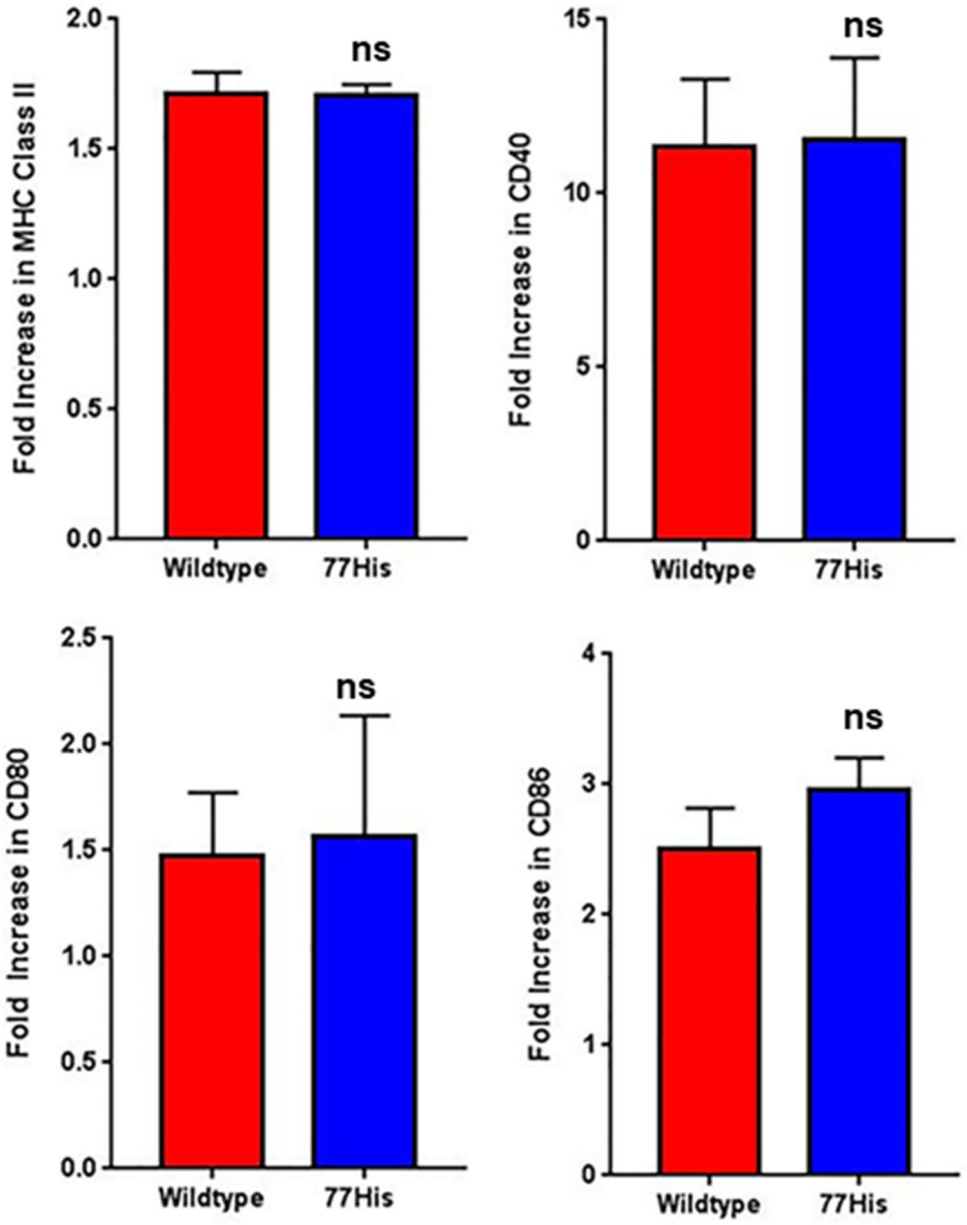
No impact of 77His on splenic DC expression of MHC class II and costimulatory molecules. Splenic DCs (CD11c^+^ cells) from 77His (*N* = 4) and WT (*N* = 3) mice were treated with 1 µg/mL LPS (serotype 055:B5) for 24 h or left untreated. The results are displayed as the fold increase in the expression of MHC class II, CD40, CD80, and CD86 (LPS/untreated)

One possible mechanism by which 77His leads to reduced T cell proliferation may involve altered expression of costimulatory molecules on DCs. Previously, ligation of Mac-1 has been shown to lead to elevated expression of MHC-II, CD86, and CD40 on DCs (Behrens et al. 2007). Thus, we next analyzed splenic DCs (defined as CD11c^+^ cells) isolated from 77His and WT mice for the expression of MHC class II, CD40, CD80, and CD86. Expression of these markers was examined under both baseline conditions and after treatment with 1 µg/mL LPS for 24 h. We observed no significant differences in expression of MHC class II or any of these costimulatory molecules either at baseline or after LPS treatment (Fig. 8). Splenic CD11c^+^CD11b^+^ cells were also interrogated for expression of the same markers, but once again, no significant difference was identified between genotypes (data not shown).

## Discussion

Genetic variants in many adhesion molecule genes have been shown to be highly associated with different inflammatory diseases (Anbarasan et al. 2015; de Lange et al. 2017; Raman et al. 2013). A significant challenge is deciphering whether any of these variants affect the expression, function, or activity of the encoded proteins and, if so, how that might contribute to increased disease susceptibility or severity of the associated diseases. GWAS have strongly implicated the SNP rs1143679 in the *ITGAM* gene, which results in an arginine to histidine change at position 77 in the extracellular domain of CD11b, with the risk of SLE, systemic sclerosis, and certain cancers (Anaya et al. 2012; Hom et al. 2008; Lenci et al. 2012; Nath et al. 2008). Functional genomic studies of transfected cell lines and primary human leukocytes have shown that the expression of 77His inhibits cell adhesion to substrates, their phagocytic capacity, and their cytokine regulation (Fossati-Jimack et al. 2013; Reed et al. 2013; Rhodes et al. 2012; Rosetti et al. 2015; Zhou et al. 2013). However, these model systems are all in vitro ones and thereby are unable to assess the effect 77His might have on the initiation and progression of immune and inflammatory processes. Further complicating the interpretation of the in vitro studies is the presence of additional *ITGAM* SNPs in high LD with rs1143679. For example, the variant allele for rs1143678, which encodes a serine at position 1146 in CD11b, has previously been shown to result in similar Mac-1-mediated defects in adhesion and phagocytosis to those reported for leukocytes expressing the 77His variant (Zhou et al. 2013). In many previous studies of 77His leukocytes, the presence/absence of 1146Ser was not specifically ascertained. Consequently, the alterations of cell functions observed in these in vitro investigations cannot be attributed to 77His per se with full confidence.

To sidestep these weaknesses, we report herein the generation of mice expressing a CD11b protein encoding histidine at amino acid position 77, thus mimicking the human rs1143679 SNP variant. Granted mice are not humans, but despite this obvious drawback genetically engineered mice have been a powerful and useful tool for addressing phenotypic changes resulting from genetic alterations present in the human genome (Ernst 2016). In this study 77His mice were found to be viable and fertile with no obvious spontaneous phenotypes, and no difference in Mac-1 expression was observed on different leukocyte subsets from 77His mice compared to WT. We did not observe any significant effects of the 77His variant on outcomes in two different models of localized inflammation (peritonitis and the dermal Shwartzman reaction), even though previous studies of CD11b null mutant mice have revealed an important contribution of Mac-1 to leukocyte recruitment and the resultant tissue damage (Hirahashi et al. 2006; Jawhara et al. 2017). This suggests that the single 77His amino acid change is not sufficient to alter these underlying Mac-1-dependent processes in vivo, underscoring that the biology of the 77His variant is not necessarily the same as that of the null mutation.

It is possible that the 77His variant does affect Mac-1′s ability to promote leukocyte recruitment and activation in vivo, but this effect is masked due to redundancies in function with other adhesion molecules like LFA-1 (Ding et al. 1999). In addition, it is possible that the 77His variant per se may not specifically modify these inflammatory processes, but other *ITGAM* SNPS may play a role. As discussed above, rs1143678 is in high LD with rs1143679 and the amino acid change resulting from this SNP has been shown to decrease both neutrophil adhesion to purified ICAM-1 and firm adhesion to stimulated endothelial cells in vitro. The development of additional lines of *Itgam* SNP variant mice will be needed to determine the impact of other CD11b amino acid substitutions on Mac-1-dependent functions.

Mac-1 expression on certain DC subsets has been shown to regulate T cell proliferation (Bai et al. 2012; Behrens et al. 2007; Ling et al. 2014; Varga et al. 2007). For example, Ling et al. (2014) previously showed that this integrin is important in LPS-induced signaling by myeloid DCs and that loss of its expression by splenic DCs can reduce T cell proliferation in co-cultures. In the studies reported here, donor OT-II T cells administered to 77His recipients injected with OVA showed a significant reduction in proliferation when compared to OT-II T cells recovered from similarly treated WT mice. We also observed significantly diminished OT-II T cell proliferation in response to OVA peptide in co-culture experiments using 77His splenic DCs or BMDCs. This outcome was also observed for co-cultures using CD11b null mutant splenic DCs (data not shown). This suggests that in vivo the 77His variant is sufficient to compromise the ability of Mac-1 on DCs (and possibly other APCs) to promote antigen-specific T cell proliferation. Previous in vitro studies using cultured murine bone marrow-derived DCs (BMDCs) showed Mac-1-mediated inhibition of APC functions (Behrens et al. 2007; Ling et al. 2014; Varga et al. 2007), so we tested if the 77His variant also compromises the ability of LPS-stimulated BMDCs to restrict proliferation of T cells in the presence of anti-CD3 and anti-CD28 antibodies. BMDCs generated from 77His and WT mice expressed CD11b equally, and after maturation in response to LPS expressed equal amounts MHC-II (data not shown). In contrast to the results of our antigen-specific T cell proliferation experiments, the 77His variant did not significantly alter proliferation of CD3/CD28-stimulated CD4^+^ T cells in co-culture assays with BMDCs (data not shown).

At this time, the mechanism by which this single amino acid substitution in murine CD11b leads to impaired antigen-specific T cell proliferation has not been identified. However this effect was not associated with diminished expression of MHC-II and costimulatory molecules by DCs, as was previously shown for cultured DCs from CD11b null mutant mice (Ling et al. 2014). It is possible that 77His compromises the ability of DCs to interact with one or more Mac-1 ligands, thereby altering DC intracellular signaling pathways and downstream expression of cytokines and chemokines involved in regulating T cell proliferation in vitro (Bai et al. 2012; Ling et al. 2014). Further investigations of the effects of the 77His variant on Mac-1-dependent inside-out and outside-in signaling pathways and cytokine expression in DCs are thus needed to determine whether they contribute mechanistically to the reduced T cell proliferation phenotype observed in these studies.

In summary, by substituting a proline at position 77 in the mouse CD11b protein with a histidine, we have developed and characterized a new line of mice expressing the human SNP rs1143679 variant allele. In two different acute inflammation models we detected no significant differences in leukocyte recruitment or tissue damage in 77His mice compared to WT, but we did observe (both in vivo and in vitro) that this variant inhibited the ability of APCs to promote antigen-specific T cell proliferation. Additional experiments using the 77His variant mice in other immune and inflammatory models are ongoing, and the results of those experiments will help determine the full impact of this solitary amino acid change in CD11b on Mac-1-dependent processes in vivo. Ultimately, it will be very informative to investigate whether 77His alters the development or progression of autoimmunity, which can be done by using various induced inflammatory disease models or by breeding 77His mice with SLE-prone strains of mice.
